# Salvage Lung Resection of Aspergilloma Mimicking Tumor Regrowth after Immune Checkpoint Inhibitor Therapy for Stage IV Squamous Cell Lung Cancer: A Case Report

**DOI:** 10.70352/scrj.cr.24-0096

**Published:** 2025-03-11

**Authors:** Takahiro Utsumi, Haruaki Hino, Yuki Takeyasu, Natsumi Maru, Hiroshi Matsui, Yohei Taniguchi, Tomohito Saito, Takayasu Kurata, Koji Tsuta, Tomohiro Murakawa

**Affiliations:** 1 Department of Thoracic Surgery, Kansai Medical University, Hirakata, Osaka, Japan; 2 Department of Thoracic Oncology, Kansai Medical University, Hirakata, Osaka, Japan; 3 Department of Pathology, Kansai Medical University, Hirakata, Osaka, Japan

**Keywords:** Aspergillus infection, pathological complete remission, immune checkpoint inhibitor, salvage lung resection

## Abstract

**INTRODUCTION:**

Recent advancements in chemotherapy, including immune checkpoint inhibitors, sometimes achieve complete remission in cases of stage IV lung cancer. On the other hand, immune-related adverse events may occur despite showing successful oncological effects. Herein, we report a rare case of a patient who underwent salvage lung resection after immune checkpoint inhibitor therapy for stage IVB lung cancer, which led to confirmed not only complete pathological remission but also complications with aspergilloma as a result of powerful effect for chemotherapy with immune checkpoint inhibitors.

**CASE PRESENTATION:**

A 71-year-old woman was diagnosed with stage IVB squamous cell carcinoma of the lung located in the right upper lobe, accompanied by distant organ metastases in the thoracic vertebrae and right adrenal gland. Because the tumor shrank after systemic cytotoxic chemotherapy plus immune checkpoint inhibitor therapy, a partial response was considered to have been achieved clinically, and chemotherapy was discontinued afterward. After 5 months, however, the primary lesion gradually regrew, and tumor regrowth was highly suspected. A bronchoscopic biopsy revealed *Aspergillus* organism infection other than lung cancer. As local recurrence could not be completely ruled out, salvage thoracoscopic right upper lobectomy with hilar lymph node dissection was performed uneventfully. The pathological diagnosis was pulmonary aspergilloma without residual cancer (pathological complete remission). After the surgery, an antifungal agent was administered for half a year and no obvious cancer recurrence or fungal relapse was detected over 1.5 years.

**CONCLUSION:**

A salvage lung resection via thoracoscopic surgery was considered a feasible procedure. However, preoperative imaging does not always provide clear evidence of residual cancer, especially after chemotherapy with immune checkpoint inhibitors, as seen in the current patient. Therefore, salvage surgery should be considered comprehensively for selected patients with downstaged or relapsed lung cancer based on close image findings.

## Abbreviations

CR
: complete response
CT
: computed tomography
ICIs
: immune checkpoint inhibitors
NSCLC
: non-small cell lung cancer
Th12
: the 12th thoracic vertebra

## INTRODUCTION

In recent years, advances in newly developed chemotherapy, including immune checkpoint inhibitors (ICIs), have resulted in complete remission in some cases of stage IV lung cancer.^1)^ On the other hand, although there are cases in which adverse events occur despite successful drug therapy, surgical treatment is considered for selected downstaged or relapsed patients based on precise diagnostic imaging.^2)^ How these drugs affect the outcome of salvage surgery is unclear owing to the small numbers of patients in previous studies on this aspect. Herein, we report a rare case of a patient who underwent salvage lung resection after chemotherapy, including ICIs, for stage IVB squamous cell lung cancer, which led to confirmed not only complete pathological remission but also complications with aspergilloma as a result of powerful effect for ICIs.

## CASE PRESENTATION

**Fig. 1:**
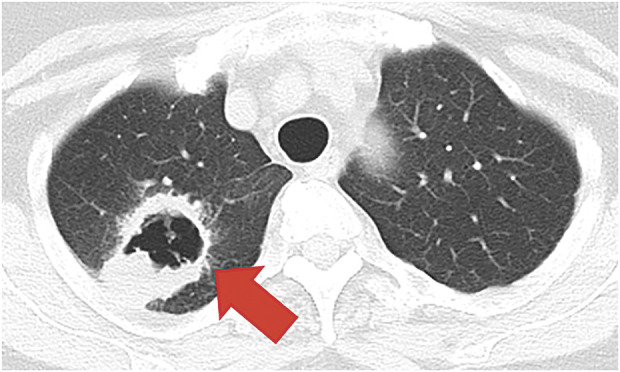
Chest computed tomography before treatment with chemotherapy and immune checkpoint inhibitor. A 31-mm mass with a cavity (red arrow) was detected in the right upper lobe of the lung. The pathological diagnosis was squamous cell lung cancer.

**Fig. 2:**
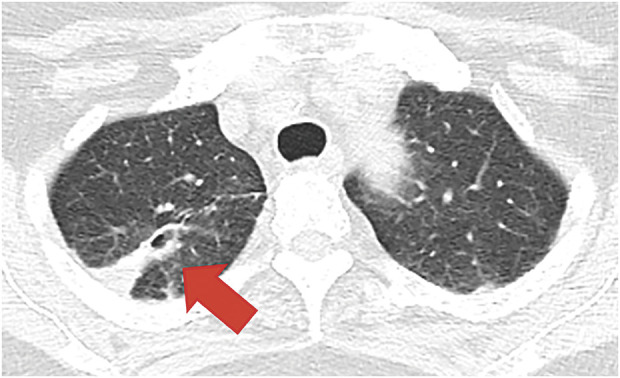
Chest computed tomography 26 months after the first treatment. The mass in the right upper lobe of the lung disappeared (red arrow); the image shows atelectasis.

A 71-year-old woman suffering from bloody sputum was referred to our hospital. Computed tomography (CT) revealed a cavitary lesion 31 mm in diameter, located in the right upper lobe of the lung, which had metastasized to the thoracic vertebrae (Th12) and the right adrenal gland (**Fig. 1**). Bronchoscopic biopsy samples led to the diagnosis of stage IVB squamous cell lung cancer, clinical stage T3N2M1c (8th edition of TNM); the epidermal growth factor mutation was “L858R,” and Programmed Death-Ligand 1 Tumor Proportion Score was unknown. After 21 courses of treatment with pembrolizumab, carboplatin, and nab-paclitaxel as first-line chemotherapy, along with palliative radiation therapy for the Th12 vertebrae (40 Gy), a partial response was observed in the primary lesion; however, the metastatic right adrenal gland continued to increase, which was estimated as progressive disease. Therefore, an additional 17 courses of docetaxel as a second line of chemotherapy were administered. During chemotherapy, no cytotoxic side effects, including bone marrow suppression or immune-related adverse events, were observed. After 26 months of chemotherapy, the cavitary lesion decreased in size to 10 mm, with shrinkage of the adrenal metastasis, as assessed on CT. We confirmed partial response based on image evaluation using the response evaluation criteria in solid tumours guideline and decided to discontinue chemotherapy (**Fig. 2**).^3)^ During long-term chemotherapy, the systemic search and size of the tumor were assessed mainly by CT scan since histological biopsy provides limited information. However, it is difficult to distinguish tumor recurrence from fibrotic and inflammatory changes on imaging.^4)^

**Fig. 3:**
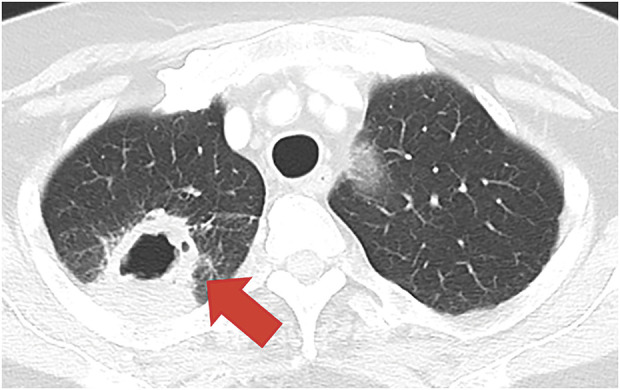
Chest computed tomography 5 months after drug discontinuation. The mass regrew to 21 mm, accompanied by cavity formation and wall thickening (red arrow).

After 5 months of observation without any therapy, regrowth of the primary lesion, up to 21 mm with cavity wall thickening, was detected on CT (**Fig. 3**). Initially, tumor regrowth was highly suspected; however, bronchoscopic biopsy did not detect malignant cells other than the presence of *Aspergillus* organisms. The patient had no fever or bloody sputum, and serum examination for *Aspergillus* antigen was negative. During preoperative discussions with thoracic oncologists, the administration of antifungal drugs with close observation was considered. However, several oncologists provided another therapeutic opinion that the regrowth on CT closely resembled a local recurrence of lung cancer with a mild, concomitant *Aspergillus* infection since cancer treatment was stopped 5 months ago. Therefore, we decided to perform thoracoscopic right upper lobectomy with hilar lymph node dissection as salvage surgery to remove the primary lesion and clarify the definitive pathological diagnosis, as well as determine the presence of residual viable cancer cells (operation time: 201 min; blood loss: 23 mL) (**Supplementary Video**). As a result, *Aspergillus* infection without any cancer recurrence was confirmed, and antifungal medicine was administered for half a year postoperatively.

**Fig. 4:**
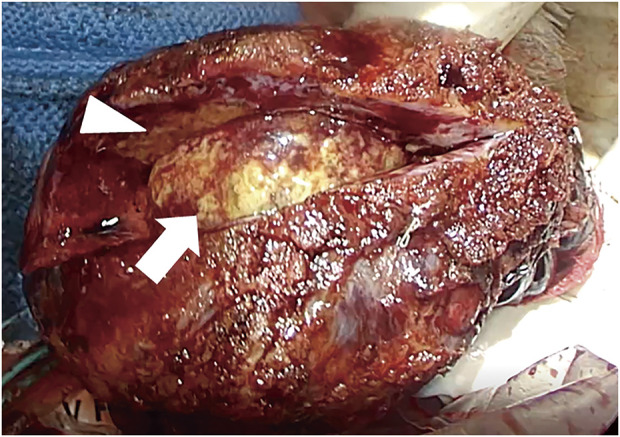
Macro image of the right upper lobe of the lung. The wall of the tumor (white arrowhead) shows thickening, and a large ball of fungus (white arrow) can be observed in the cavity. The pathological diagnosis was pulmonary aspergilloma, and no residual cancer was detected.

Intraoperatively, the hilar peribronchial lymph nodes (#11s), to which the cancer had metastasized before chemotherapy, showed severe fibrotic changes with surrounding tissue adhesion, making dissection difficult. Alternatively, dissecting the perivascular sheath on the central side of the pulmonary artery, which was not invaded by the tumor, enabled the detection of a normal anatomical structure. The pulmonary vein, artery, and right upper bronchus were dissected in order using linear stapler (Endo GIA Ultra Universal stapler short; Medtronic, Minneapolis, MN, USA). Finally, the interlobar fissure was dissected to reduce postoperative air leakage, which is referred to as fissureless lobectomy. Lymph node dissection of the hilar peribronchial node was performed as much as possible since the node itself had changed to fibrotic and granular tissue. After lung resection, a large fungal ball was observed in the cavity of the excised right upper lobe (**Fig. 4**). The final pathological diagnosis was pulmonary aspergilloma, with no residual cancer in the resected right upper lobe nor hilar lymph nodes (pathological complete remission).

The patient was discharged from the hospital on the 8th postoperative day without any complications. After receiving antifungal medication for half a year postoperatively, the patient showed no recurrence of cancer or relapse of *Aspergillus* infection for over 1.5 years without any anticancer chemotherapy.

## DISCUSSION

Newly developed chemotherapy agents such as ICIs are effective in patients with advanced or recurrent non-small cell lung cancer (NSCLC). Moreover, the efficacy of nivolumab as neoadjuvant therapy for patients with surgically resectable NSCLC (CheckMate 816) has recently been reported.^5)^ The uptake of salvage surgery is also increasing owing to the success of these types of chemotherapy, resulting in favorable outcomes regarding local control in selected patient groups.^1,6)^ According to Smith et al.,^7)^ in 9 cases of surgery after chemotherapy with ICI, no postoperative hospital deaths were reported. However, no official guidelines are available on the indications, optimal procedure, and postoperative treatment of salvage surgery, making it difficult to select suitable patients.^8)^ According to 7 case series recently reported on salvage surgery after chemotherapy with ICIs for unresectable advanced lung cancer, we reviewed surgical results of 99 patients, including those studies.^1,9–13)^ Minimally invasive surgery and thoracotomy were performed in 27.3% (27/99) and 72.7% (72/99) of patients, respectively. The conversion rate of minimally invasive surgery to thoracotomy was 37.0% (10/27 cases). The reason for thoracotomy was that the excellent antitumor effect of the ICIs led to not only tumor shrinkage but also severe fibrosis in neighboring lesions, making the surgical technique rather complicated.^14)^ In contrast, some patients who had less invasive cancers or discrete lymph node metastasis, such as our case, did not have totally severe inflammation and fibrosis in the hilum.^15)^ Therefore, salvage surgery after treatment with ICIs could be performed safely via minimally invasive surgery, especially for such cases. Postoperative complications and 90-day mortality were observed in 31.3% (31/99) and 4.0% (4/99) of cases, respectively, which was considered an acceptable result. Moreover, complete pathological response was achieved in 23.3% (10/43 cases) of patients. The 2- and 3-year overall survival rates were 76.2%^10)^ and 35.9%,^11)^ respectively, and the total observation time was 8.3–17.4 months, which is considered immature data and expected for long-term observation. Since surgeons have limited experience with salvage lung resections in patients treated with ICIs, a larger number of surgeries is required to elucidate indications, timing, approach, procedure, postoperative treatment, and survival time in such cases.

This case was a rare presentation of aspergilloma in a patient with stage IV lung cancer after treatment with cytotoxic chemotherapy using ICIs following salvage lung resection. Particularly, we discussed a treatment strategy in the cancer board session. Apart from emergency salvage surgery for such a critical empyema or bleeding conditions,^2,16)^ the preoperative CT image showed a closely mimicking regrowth of the primary tumor rather than aspergilloma without any symptoms. Furthermore, the bronchoscopic biopsy could not completely rule out local recurrence of lung cancer, and the patient had no fever or bloody sputum, with negative serum *Aspergillus* antigen. Although the preoperative bronchoscopic biopsy revealed the presence of *Aspergillus*, conservative observation with antifungal drug administration was another therapeutic option. However, we decided to perform a lung resection before it grew any further not only for obtaining a correct histological diagnosis but also for treating a residual region of cancer or fungal infection. As a result, aspergilloma was pathologically confirmed postoperatively, and subsequent antifungal therapy was completed without any adverse events.

Postoperatively, a discrepancy between preoperative imaging and postoperative pathological diagnosis was confirmed, representing the significant impact of ICIs, which was more than we estimated preoperatively. Thus, especially for preoperative imaging, the response evaluation criteria in solid tumors may not be accurate for evaluating the efficacy of ICIs in the case of “pseudo-progression,” which should be carefully considered.^3,17)^ According to a previous case report of a 57-year-old male patient, chemotherapy with ICI for postoperative local and distant organ recurrence of pleomorphic carcinoma after left upper lobectomy showed a typical characteristic of “pseudo-progression.”^18)^ An autopsy revealed that viable cancer cells of local and adrenal gland recurrence were <5%, and most of the remaining tissue consisted of degenerative, necrotic, and scar tissue with moderate-to-severe inflammatory cell infiltration. Thus, tumor regrowth during chemotherapy with ICIs observed on CT may incidentally be as a “pseudo-progression.” Therefore, the pathological diagnosis after chemotherapy with ICIs should be clarified by salvage lung resection when cancer invasion is limited. Nonetheless, further experience with such salvage cases is required to clarify the precise therapeutic effect of ICIs on preoperative imaging and surgical and pathological outcomes.

Lung cancer and pulmonary *Aspergillus* are occasionally diagnosed simultaneously.^19)^ The detection rate of pulmonary *Aspergillus* from sputum or bronchoalveolar lavage fluid is relatively low, at approximately 40%–50%.^20)^ Therefore, multidisciplinary approaches using CT and serological examinations are recommended for the diagnosis of pulmonary *Aspergillus*.^21)^ The causes of *Aspergillus* engraftment in patient with lung cancer are considered as follows: a decrease in local immune function, underlining condition of chronic obstructive pulmonary disease and interstitial pneumonia, and cavity formation where cancer was growing and disappeared after effective cytotoxic chemotherapy with ICIs and/or radiation therapy. In this case, although preoperative imaging demonstrated a mimicking of local recurrence of lung cancer, salvage surgery could obtain a pathological diagnosis of no residual tumor (pathological complete response [CR]) and curability for aspergilloma, which was an unusual presentation. Therefore, we should consider both cancer curability and opportunistic infection in such a case as a differential diagnosis.

Although a few surgical experiences have been reported, salvage lung resection via thoracoscopic surgery for advanced lung cancer after chemotherapy plus ICIs seems feasible; however, dense hilar fibrosis may make such surgeries challenging.^15)^ Furthermore, preoperative imaging does not always provide clear evidence of residual cancer in such cases. In our case, we intraoperatively devised a surgical technique for vulnerable lungs after chemotherapy; we performed right upper lobectomy, primarily resecting the hilar components of the pulmonary vein, artery, and bronchus, and finally dissecting the interlobar fissure complicated by an incomplete lobulation, which was called “fissureless lobectomy”^22)^ (**Supplementary Video**). Additionally, the hilar lymph nodes, which were swollen due to cancer metastasis prior to treatment, presented as fibrotic tissue with partial necrosis and adhered to the pulmonary artery and bronchus after chemotherapy. Thus, highly technical skills for lymph node dissection via thoracoscopic surgery are needed. Fortunately, we detected an intact part of the pulmonary artery and bronchus apart from the previously metastatic lymph nodes and were able to expose a normal anatomical structure connected to the right upper lobe. Alternatively, an additional procedure with pulmonary artery plasty through clamping of the central and peripheral sides of the main pulmonary artery was considered. Therefore, a flexible response for surgical technique based on cancer invasion, scar, and fibrosis was required, especially for salvage surgery after chemotherapy with ICIs and/or radiation therapy. Through our procedures, we should refrain from manipulating cancer-invaded tissue where a normal anatomical structure cannot be visually ascertained. Thus, surgical technique is considered an important core maneuver and is essential for performing a safe procedure in salvage lung resection.^12)^ Accordingly, salvage surgery should be considered in carefully selected patients with downstaged or relapsed lung cancer after ICIs, with sufficient preoperative consideration of the surgical process and approach. A larger number of salvage surgeries, especially following chemotherapy with ICIs, along with consultation with an oncologist for each patient with different cancer locations and variations of chemotherapy and/or radiation therapy, may clarify its utility and survival benefit in the future.

## CONCLUSION

A salvage right upper lobectomy via thoracoscopic surgery for suspected lung cancer recurrence (originally clinical stage IVB squamous cell carcinoma) after chemotherapy with ICIs was considered a feasible procedure with appropriate patient selection. As a result, pathological CR of lung cancer complicated with aspergilloma located in the primary cancer growth was ascertained. However, the precise assessment of the therapeutic effect of ICIs on preoperative imaging is challenging. Additionally, surgical and pathological outcomes, and long-term survival of salvage lung cancer resection remain unclear. Therefore, further experience in salvage thoracoscopic surgery for lung cancer pretreated with ICIs is needed. Multi-institutional cohort studies for such cases are desirable to prove the significance of this procedure in the future.

## SUPPLEMENTARY MATERIALS

Supplementary Video Thoracoscopic right upper lobectomy as a salvage surgery.

## ACKNOWLEDGMENTS

The authors would like to thank Editage (https://www.editage.jp) for the English language review of the manuscript.

## DECLARATIONS

### Funding

Not Applicable.

### Authors’ contributions

TU: conceptualization, data curation, investigation, operational performance, and writing of the original draft.

HH: operation and data curation.

YT and TK: treatment.

NM, HM, YT, and TS: data curation.

KT: pathological diagnosis.

TM: supervision.

All authors reviewed and approved the final version of the manuscript and had accountability for all aspects of the work.

### Availability of data and materials

The datasets supporting the conclusions of this article are included within the article.

### Ethics approval and consent to participate

This work does not require ethical considerations or approval. Informed consent was obtained for this case report.

### Consent for publication

Written informed consent was obtained from the patient for the publication of this case report and any accompanying images.

### Competing interests

Takayasu Kurata received honoraria from MSD, Ono, Chugai, Nipponkayaku, Bristol Myers Squibb, Eli Lilly, AstraZeneca, Pfizer, Takeda, Janssen, Novartis, Boehringer Ingelheim, Amgen, and Daiichi Sankyo. The other authors declare that they have no competing interests.
